# Optical Angular Sensor for Space Applications

**DOI:** 10.3390/s21175979

**Published:** 2021-09-06

**Authors:** Alexander Dabsch, Christoph Rosenberg, Majesa Trimmel, Franz Keplinger

**Affiliations:** Institute of Sensor and Actuator Systems, University of Technology Vienna, 1040 Vienna, Austria; christoph.rosenberg@tuwien.ac.at (C.R.); majesa.trimmel@tuwien.ac.at (M.T.); franz.keplinger@tuwien.ac.at (F.K.)

**Keywords:** angular sensor, optical sensor, self-referencing, Si-based-sensor

## Abstract

This paper describes a silicon/glass sensing structure for axial angle measurements. The presented optical angular sensor can statically measure the angle *φ* of any apparatus depending on the torsion of the optical component against the sensor housing. Core element of the sensor is an optical medium with an etched structure, which diffracts light from an LED according to the Fresnel equation. Two photodiodes, one for angle determination and one as reference, conduct the measurement. Hence, the signal splits up into two parts: one part transmits trough the optical system and the second part (the reflected wave) is used as reference signal. For self-referencing purposes, the wavelength spectrum of the LED has its maximum in the infrared regime near to the wavelength where silicon gets transparent (l~1000 nm). More precisely, torsion angle and light intensity show a dependency given by $T_{s_{tot}}$ if a straight etching structure (refraction profile) is used. To avoid multiple reflections of light, a coating layer restricts the illuminated area in the optical medium. With this setting a resolution of 0.05-degree rotation angle has been achieved and by stacking the construction, the sensor can measure an angular range from 30° up to 270°.

## 1. Introduction

Angular sensors are of great significance for the aerospace industry. However, there are no standard sensors that are otherwise specified by ESA or NASA due to extremely high requirements for operational reliability and necessary redundancy.

Commonly used measuring components are either capacitive, inductive or resistance dependent sensors for static measurements or incremental encoders, which work optically through shading (light barrier principle) [1]. The advantages of these measuring principles are the simple structures and low manufacturing costs. Nevertheless, the outcoming results depend heavily on external factors. The use of magnetic sensors is therefore ruled out in many applications where external magnetic influences are present and would inadmissibly falsify the measurement result. The downside of other alternative systems lies in the necessary referencing (movement path, reference position, etc.) and the energy supply fluctuations, thereby creating the need for a second simultaneously used sensor for a redundant system. The use of such systems is correspondingly expensive. In addition, the space requirement increases and thus prevents the miniaturization desired for many applications.

Satellites in which several such sensors are incorporated steer by tilting one of their engines relative to their center of mass to generate a torque. For this, the mechanism will be reactivated after the launch procedure has finished. Conventional systems perform a reference drive from one dead center to the other to determine the correct position. To increase the energy-efficiency of the engine control, the proposed self-referencing angular sensor displays the correct position without a reference drive [2]. Due to the optical principle, the component is independent of E- and B-fields while also being cost-effective.

The presented demonstration object is the first step towards an industrial prototype. Convincing results have already been obtained with little effort and are fully comparable to the computations of the project. Current challenges that need to be addressed in order for the sensor to reach its full potential as well as future prospects will be discussed in this paper.

## 2. Materials and Methods

The idea behind the sensing element is based on the fundamental equation of optics [3]. The angle-dependent transmittance of a bundled light ray trough a suitable optical medium and the related boundary surfaces are given by:

(1) $${\left(\frac{E_{0t}}{E_{0e}}\right)}_{s}=T_{s}=\frac{2N_{1}\cos\alpha }{N_{1}\cos\alpha +N_{2}\cos\beta }$$

**Figure 1:**
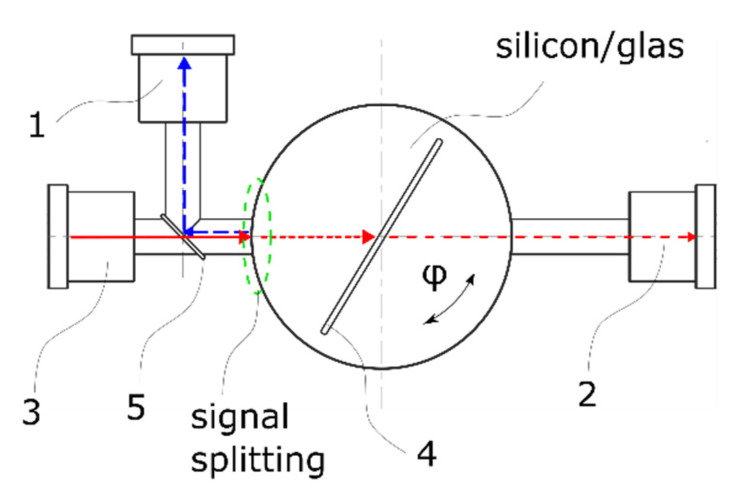
Sensor principle comprising: (1) reference photodiode, (2) measurement photodiode, (3) LED, (4) optical medium with etched structure and (5) semitransparent mirror.

With $T_{s}$ as the transmission, $N_{i}$ are the refractive indices of the optical medium and its inner structure whereas α and β are the angles of incidence at the entry and exit of the light beam. The index s represents the perpendicular polarized light. For the applied design α is equated with φ (see Figure 1) and β is the result of Snell’s law.

(2) $$N_{1}\sin\alpha =N_{2}\sin\beta$$

As shown in Figure 1, the beam path has to cross two boundary surfaces and the air gap formed by the etched structure to determine the wanted angle. After the entry of the optical medium at an angle of 90° the ray hits the etching structure with an intensity of $I_{max}$. Due to the differing refractive indices the law of optics applies and leads to a small offset compared to the initial entrance beam. Thereby the LED’s output intensity characertiscs and maximum $I_{max}$, which are similar to the entrance intensity characteristics of the photo diode, shift depending on the rotating angle of the structure. Consecutively, the photo diode’s absorption capacity is dampened and can be utilized to determine the wanted angle φ. The exit angle of the beam path is thereby nearly parallel to the input beam path with 90° − β since β can be ignored due to its low value (β << 1). Subsequently the beam path passes through the exit of the optical medium consisting of a black aperture with an opening of 1 mm which therefore absorbs all other emerging internal noise reflexions.

The rotating optical medium is made of a first material having a refractive index $N_{2}$. It is intended that the rotating element contains at least one refractive structure having a second refractive index $N_{2}$ with the condition $N_{1}\;\neq \;N_{2}$. Preferably $N_{1}<N_{2}\;$ and a symmetrical design also apply to the system [4]. Therefore, the shape and the arrangement of the structure, especially in a plane in which the light generator and the detector lie, determine the signal as a function of the angle of rotation φ.

To simply produce the refractive design with an index $N_{1}$ it is provided that the structure is formed by a recess in the first material. The established method offers a particularly high accuracy as well as high reproducibility for the production of the sensor and ensures that at wavelengths greater than those in the X-ray range the condition $N_{1}<N_{2}$ will be met. Furthermore, very small refractive structures can be produced in a defined manner, possibly also in the micrometre or submicrometer range, which favors the miniaturization of the invention. It should be emphasized that a refractive structure need not be formed as a recess. The surrounding material could also be doped to manufacture the second structure.

Using well-established etching processes for the desired refractive structure yields a recommendation for the surrounding material to be either silicone or glass [5]. For this purpose, silicone material means Si-based components, in particular Si-wafers and -substrates and analogously the same applies to glass. By appropriate selection of the medium, the refractive indices can be varied in a targeted manner, which—in addition to the geometry of the structure—can be used to design the transmission function. Moreover, silicone as a clean alloy results in a deformation, temperature and aging resistant material due to its single crystal nature.

**Figure 2:**
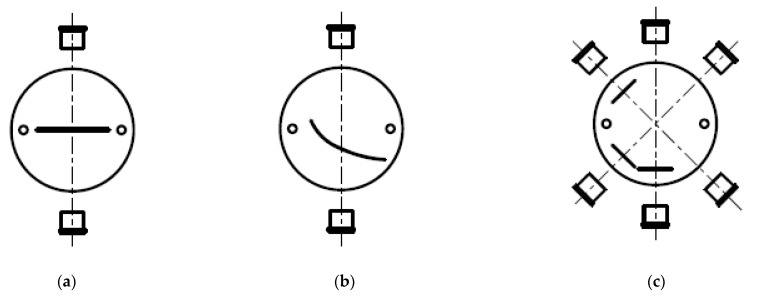
(**a**) Straight gap with nonlinear behavior; (**b**) Precisely defined curve shape for linear behavior; (**c**) Stacked construction for extended range.

To further ensure a certain redundancy of the measurement with only one sensor, it is preferred that the rotating element is made up of several layers with at least one refraction structure provided in each of these. Additionally, the measuring range can therefore be extended from approximately 30° to more than 270° by arranging the structures in the element in a certain angle from each other. Aiming to obtain a wider measuring range through such stacking, the different layers are situated in such a way that the beam paths run through the structures in differing angular ranges (Figure 2c). These sections overlap ideally so that the resulting measuring range is continuous and has no gaps. A limit of 270° has been manually set as no apparatus is incorporated to detect a full rotation and due to the occurrence of total reflexion and increased multiple noise reflexions.

It is intended that at least one light source and at least one light detector are arranged opposite each other (Figure 2a,b). The type of detection is technically particularly easy to realize, whereby a wealth of detectors known per se can be used as means (e.g., photodiode). Favorably, laser diodes or LEDs, which allow downsizing of the sensor, can be used as light sources. The selection should however still be made regarding the wavelength range used, but it is not necessary that the generated light must lie in the optically visible wavelength range. To easily distinguish signals from different light-generating means, the components should be designated for different wavelengths.

As a means to avoid fluctuations due to the temperature-dependent power supply of the light source, at least one reference LED is provided with the intention to catch the emitted light before it hits the refraction structure. The measurement leads to the interception of altering the light by the optical medium and the light can thus be set in relation to the measurement result. This allows one to prevent and compensate variations in the properties of the emitted light beam.

The complete reduction of the transmitted intensity by the passage is described by the following equation:

(3) $$T_{s_{tot}}={\left(\frac{2N_{2}}{N_{1}+N_{2}}\right)}^{2}\left(1-\frac{N_{2}\cos\alpha -N_{1}\cos\beta }{N_{2}\cos\alpha +N_{1}\cos\beta }\right)\left(\frac{2N_{1}\cos\beta }{N_{2}\cos\alpha +N_{1}\cos\beta }\right)$$

The first term represents the reduction through the rectangular invasion at the entrance and the exit of the wafer, respectively. The remaining two terms represent the pass through to the etching structure, with the assumption of an optical thinner medium in the gap (e.g., air). To consider the absorption of the material, $T_{s_{tot}}$ is multiplied by $e^{-aD}$ with a as the wavelength dependent absorption coefficient and D as the diameter of the silicon wafer [6]. The absorption coefficient a includes the extinction coefficient n*”* and depends on the wavelength ω of the high ray.

The transfer function is essentially determined by the geometry of the refractive structure. However, a simple geometry does not necessarily result in a simple functional relation between the property of the detected light and the angle of rotation. Transmission resulting for a rectilinear refractive structure is for instance nonlinear. If a linear or other desired function for the transmission is wanted (e.g., phase shift), the geometry can be recalculated or adapted by means of simulation.

One of the preferred materials of the optical medium is silicon. The following calculations were therefore carried out with the intent of finding the transmission function.

Si has a refractive index of $N_{2}=3.542$ at a wavelength of 1100 nm [7] while the maximum index of 6.891 can be found at 370 nm. Furthermore, it can be assumed that the refactive index at the used wavelength stays approximately the same under the lower temperature conditions in space [8]. The index of the refractive medium $N_{1}$, in this case air, is 1 and the width of the gap was defined as 0.1 mm due to the maximum of the rotation sensitivity encountered there. The parameters are also listed in Table 1.

The calculation provides the expected transmission in respect to the rotation. For this execution, the absorption coefficient of the medium in the gap is neglected, taking the thin etching structure into consideration. In addition, the displacement of the light beam after passing through the gap is overlooked due to the wide recording surface of the photodiode.

**Figure 3:**
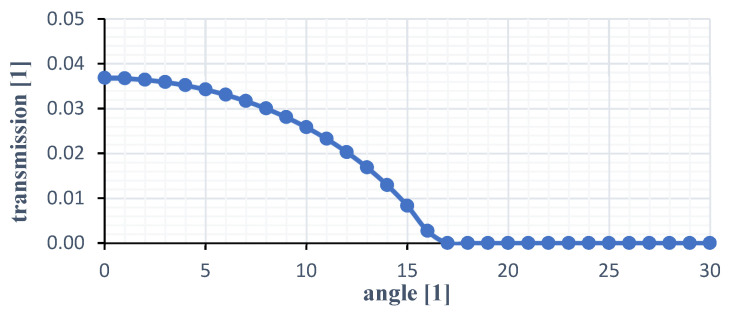
Transmission curve for silicone as optical medium.

The transfer function pictured in Figure 3 depicts the logarithmic ratio between twist angle and transmission with the cut off at 17° (depending on $N_{1}$ and $N_{2}$) by total reflection. The first angle of rotation range ends here at the latest.

The calculations were also performed for glass whose parameters can be seen in Table 2 below.

**Figure 4:**
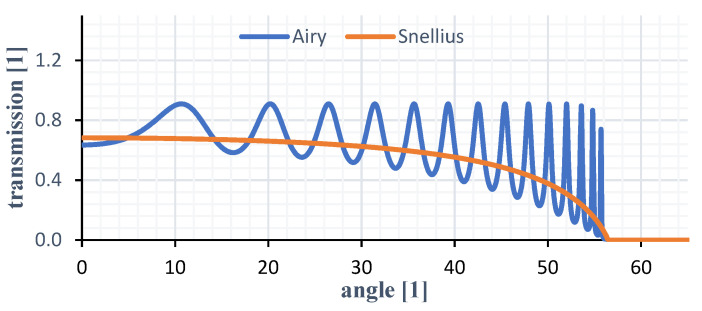
Transmission curves for a sensor with and without coating with glass as optical medium.

The cutoff here takes first place at an angle of 56°. As already known, these measurements are based on the angle dependent transmittance of the structure. Additionally, an anti-reflexion coating within the structure can avoid making multi-reflexions occur. Figure 4 shows the comparison between the Airy reflexion—multi-reflexion without coating—and the Snellius equation with an optical gap of 0.005 mm. The latter equals the simulated transfer function for the glass material with the specified gap width.

## 3. Measurement

**Figure 5:**
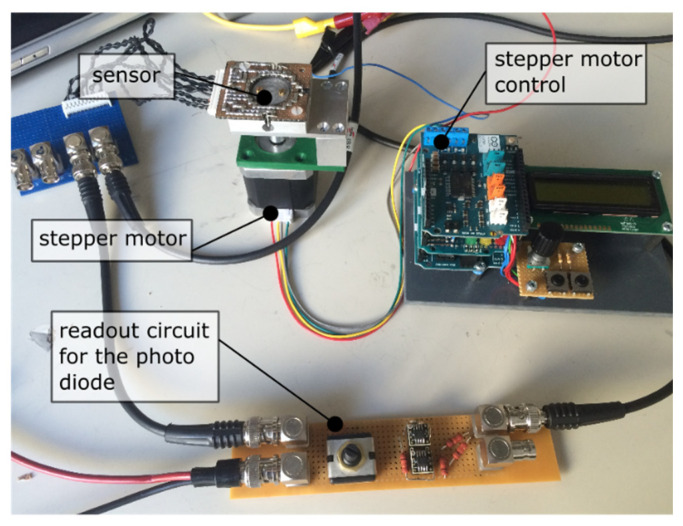
Measurement setup with stepper motor control panel and photodiode readout circuit.

To verify the findings, a simple device was built with a stepper motor fixed directly to the rotatable part of the sensor without a gearbox. However, the optical medium here was made from acryl glass without extra coating for the time being because of simpler manufacturing. Without a bearing of the medium in the sensor housing we expected a minor inaccuracy during the measurement due to the smaller positioning accuracy (Figure 5).

**Figure 6:**
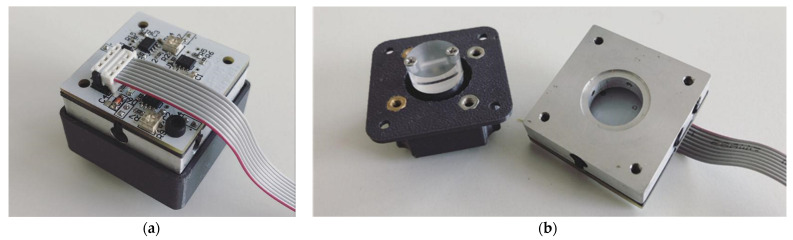
(**a**) Sensor board integrated in 3D printing case; (**b**) Main section of the sensor with all optical components.

Due to the control panel of the stepper motor, it is possible to drive and hold single steps with a resolution of 0.9 degree. An angle range from 0° to 90° has therefore been surveyed and repeated. The readout was realized with two OPA129 operational amplifiers and a controllable measuring current (see Figure 6). A function generator (Agilent 33220A) drives the laser diodes. The electrical parameters are listed in Table 3.

Furthermore, an additional reduction of the transmission was expected due to diffuse light reflection on the rough surface caused by the milling process.

The following figures show the current design of the sensor in a 3D printed enclosure with circuit and optical components already integrated.

## 4. Results and Discussion

**Figure 7:**
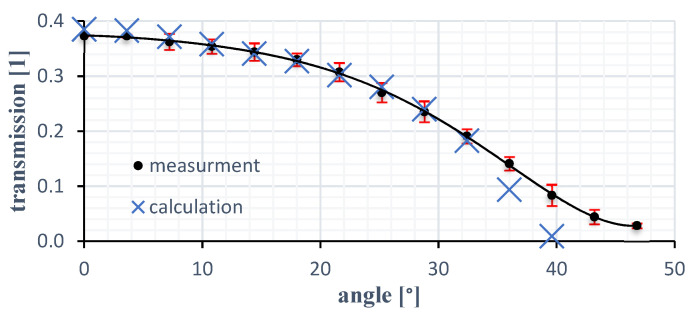
Measured transmission characteristics compared to the computed characteristics with acryl.

**Figure 8:**
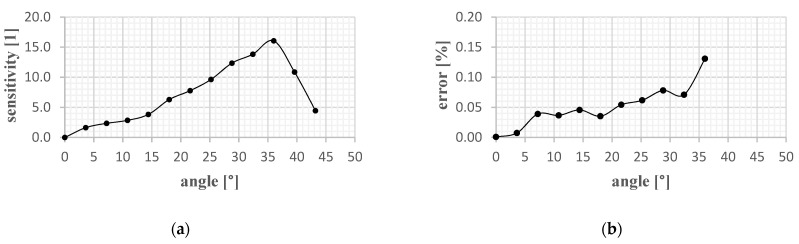
(**a**) Determined sensitivity for the measured transmission; (**b**) Determined error rate for the measured transmission.

The data acquisition was organized with a lock-in-amplifier (SR830). To quantify the repeatability ten measurements per step were performed. The results for the sensor with an optical medium of acryl are depicted in Figure 7 with an enlarged (10×) standard deviation. The average standard deviation is 0.012 which is equivalent to approximately 0.25° and an error rate of 3.2%. It should be noted that these measurements only apply up to 30° (see Figure 8).

Due to the wider gap and the milled surface, total reflection occurred earlier than the calculation had predicted. The lack of the discussed coating also influences the transmission and increases the reflectivity of the optical interfaces. The reference diode signal is thereby proportional to the input voltage, but for ease of comparability, the quotient between input voltage *U_ss_* and output voltage after amplification is shown.

The measured results are in line with the computed transmission behavior and the achieved measuring range is comparable with the theoretical mode. Furthermore, the results obtained here are comparable to other angle sensors that work with similar principles. According to Cavallo et al. [9] the optoelectronic joint angular sensor has an error of 2.5% over an angular range of 90° and a maximum deviation of 1.8%.

A detailed comparison of the existing principles with the presented sensor can be taken from Table 4. Space projects are primarily concerned with position detection on slewing gears on engines of large satellites (research/telecommunications). A prerequisite for this application is that the sensor is mounted directly concentric with the center of rotation (for space reasons). This design requirement already eliminates some competing principles which would meet the other requirements. Furthermore, a contactless measurement of the angle is required for a range of at least 30°. The argument of self-referencing (switch-on accuracy) is not mandatory for the function but represents an enormous advantage for the energy efficiency of the entire system and due to strict ESA guidelines, the required E- and B-field compatibility makes some principles difficult to implement. Hence, optical sensors represent the optimal solution. Another argument for the comparison of existing technologies with the newly presented sensor is the longevity with regard to the contamination of the optical components, which is why the use of an optical moving medium with only low aging properties is preferred. The discussed sensor combines the desired properties and is therefore well suited for applications in space.

## 5. Conclusions and Outlook

In this paper, an optical angular sensor based on the fundamental law of optics was proposed and discussed. The optical sensor principle developed uses the angle-dependent transmission of a light beam with a defined wavelength at an optical interface between materials with different refractive indices. A defined trench is created in a disk (e.g., SOI-wafer by chemical etching processes) which is illuminated from different directions by LEDs. Depending on the twisting angle of the wafer, multiple refractions of the light beam occur, which allows the exact position to be determined by multiple photodiodes. By embedding several photodiodes in a stacked or even integrated array, the range of the measurable angle can be increased significantly from 30° up to 270°.

The result obtained from the experiment with a prototype shows that the transmission characteristics are fully comparable to the computational and theoretical models of the sensor. Additionally, in contrast to the available conventional sensing methods the sensor has the advantage of an optical measurement, self-reference, and a wider measuring range. Hence proving that it is well suited for space applications.

By implementing an SOI-wafer and wet a chemical etching process in combination with optical coatings (e.g., zinc selenide) an improved version of the prototype is planned. The sensor will therefore be manufactured with an optical medium made of Si or glass and is to be downsized to further confirm the obtained results.

Moreover, a stacked assembly with at least three sensing elements is part of a further research project with the aim to get a self-referencing sensor for a 120° measuring range.

A second objective may be to develop an alternative structure with a linear transmission behavior to increase the measuring range for a single stack sensor. For this, a logarithmic formed etching gap is placed eccentrically on the SOI-wafer and a second laser diode/photo diode couple shifted by 90° with another nominal wavelength operates in parallel for a redundant measurement.

## 6. Patents

The proposed invention has already been patented and can be found under the publication number AT517945A4, AT517945B1, CA3005550A1, DK3377860T3, EP3377860A1, EP3377860B1, US10845217B2, US2018372515A1, or WO2017083898A1.

## Figures and Tables

**Table 1:** Parameter for silicone as an optical medium.

Symbol	Quantity	Value
N_1_	refractive index 1 (air)	1
N_2_	refractive index 2 (silicon)	3.542
d	Gap width	0.1 mm
D	Diameter of the optical medium	3 mm
a	Absorption coefficient	1 cm^−1^

**Table 2:** Parameter for glass as an optical medium.

Symbol	Quantity	Value
N_1_	refractive index 1 (air)	1
N_2_	refractive index 2 (silicon)	1.2
d	Gap width	0.1 mm
D	Diameter of the optical medium	3 mm
a	Absorption coefficient	1 cm^−1^

**Table 3:** Electrical parameter used in the experiment.

Symbol	Quantity	Value
U_ss_	input voltage	450 mV
U_o_	offset	1.85 V
V_pp_	power supply op-amp	10 V
f	frequency	5 kHz

**Table 4:** Comparison of different angular sensors.

Paper Reference	Contactless	Self-Referencing	Range
Proposed sensor OASIS	Yes	Yes	35° (Single) 270° (Stack)
Optoelectronic joint angular sensor for robotic fingers [9]	Yes	No	90°
Nanoradian angle sensor and in situ self-calibration [10]	Yes	No	0.03°
High-sensitivity small-angle sensor based on surface plasmon resonance technology and heterodyne interferometry [11]	Yes	No	0.3°
A precision angle sensor using a multi-cell photodiode array [12]	Yes	Yes	0.6°
A novel silicon surface micromachining angle sensor [13]	No (Lorentz force)	No	180°
360° angle sensor using spin valve materials with SAF structure [14]	No (magnetic)	No	360° (Stack)
Angular position sensor for space mechanisms [2]	Yes	No	360°
Magnetic angular position sensor enabled by spin-orbit torque [15]	Yes	No	360°
A Flexible, Planar-Coil-Based Sensor for Through-Shaft Angle Sensing [16]	Yes	No	90° (Single) 360° (Stack)
Immersion-type KTP sensor for angular displacement measurement [17]	Yes	No	10°

## Data Availability

Mail to alexander.dabsch@tuwien.ac.at.
